# Preface to ‘The future of mathematical cosmology'

**DOI:** 10.1098/rsta.2022.0002

**Published:** 2022-05-02

**Authors:** Spiros Cotsakis, A. P. Yefremov

**Affiliations:** ^1^ Institute of Gravitation and Cosmology, RUDN University, ul. Miklukho-Maklaya 6, Moscow 117198, Russia; ^2^ Research Laboratory of Geometry, Dynamical Systems and Cosmology, University of the Aegean, Karlovassi 83200, Samos, Greece

This is the first part of the theme issue ‘The Future of Mathematical Cosmology' which is published in two separate volumes by the *Philosophical Transactions of the Royal Society A*. Overall, the two volumes contain a number of research, review and opinion articles, covering multiform aspects of mathematical and theoretical cosmology.

The subject of mathematical cosmology plays a fundamental role in theoretical physics today and has deep applications in contemporary astronomy and astrophysics. Motivating important new ties and connections with general relativity and correlating with diverse features of string theory and quantum gravity, theoretical cosmology is in fact the most obvious ‘testing agent' for many of the most advanced, pioneering or speculative ideas in these fields.

Mathematical cosmology has acquired a unique status, potential and independence as a scientific field. It reveals its own problems, methods and techniques, often with the stimulus of general relativity, quantum field theory and above all differential geometry, but it also serves as the inevitable tank of ideas and models for use of the more data-driven, ‘actual' physical cosmology. In fact, mathematical theoretical cosmology provides the driving force behind modern attempts to explain the—now manifold—observational cosmology data.

We believe that this theme issue will be useful as a lasting guide and reference for interested people entering the field, as well as for experienced researchers who seek to expand their field of vision in this most majestic of scientific enterprises.

We are grateful to the Royal Society for inviting us to guest-edit this theme issue, and especially to its Commissioning Editor, Ms Alice Power, for her precious help offered to us so willingly in all stages of this demanding project. We are also grateful to all contributors of this theme issue—old and new friends—who agreed to participate in this project and succeeded in writing such interesting, unique and novel articles covering a huge amount of cosmological ground. The fact that mathematical cosmology is a time-honoured field but at the same time such a timely and lively subject is testimony to the papers included in this theme issue and the efforts of all those devoted individuals who made that possible.

## Data Availability

This article does not contain any additional data.

